# The contribution of planted forests to regional carbon storage: evidence from western Hunan, China (1990–2020)

**DOI:** 10.1186/s13021-026-00443-3

**Published:** 2026-04-20

**Authors:** Ting Deng, Yulin Zhu

**Affiliations:** 1 School of Ecology and Environment, Central South University of Forestry and Technology, Changsha, 41004 China; 2 College of Economics and Management, Central South University of Forestry and Technology, Changsha, 41004 China

**Keywords:** Planted forests, Natural forests, Carbon sequestration, Land use conversion

## Abstract

The widespread expansion of planted forests, fueled by initiatives such as China’s Grain-for-Green Program, plays a critical role in climate mitigation, yet its carbon performance compared to natural forests remains contested. Using​ spatiotemporal analysis, this study systematically ​examines​ the dynamics of planted and natural forests in the western region of Hunan from 1990 to 2020, and ​assesses​ their impacts on regional carbon storage. The results reveal a marked forest transition: while natural forests declined, planted forests expanded considerably, creating a distinct spatial trade-off—planted forests proliferated mainly at mid-low elevations (300–700 m), whereas natural forests were largely confined to higher elevations (400–900 m). Although natural forests stored more carbon overall (55.1% of the regional total), planted forests exhibited superior carbon sequestration rates per unit area.​​ Notably, newly established planted forests on converted land accumulated almost equivalent carbon stocks (19.0 Tg C) to older, stable natural forests (19.5 Tg C), highlighting their rapid carbon uptake potential. Spatiotemporal analysis confirmed that ​conversion of cropland was the dominant pathway, driving both the expansion of planted forests and the associated carbon gains. These findings ​quantify the net carbon gain from restoration policies​ and underscore that future land-use strategies must ​manage their spatial trade-off​ to secure long-term climate and ecological benefits.

**Supplementary Information:**

The online version contains supplementary material available at 10.1186/s13021-026-00443-3.

## Introduction

​ The expansion of planted forests has been widely promoted as a key strategy for enhancing carbon sequestration and achieving ecosystem restoration goals globally [1–3]. However, ​a consensus is lacking on whether these expansions represent a net gain in carbon storage, especially when they replace natural forests or are established on non-forested lands [4]. This controversy stems from a scarcity of studies that simultaneously track forest type dynamics and their carbon outcomes over space and time [2]. China’s forest transition provides a critical case study: from 1990 to 2020, the nation’s planted forest area increased by approximately 447,500 km², while natural forests declined by 219,100 km² [5]. Consequently, our study aims to clarify the contributions of both forest types by quantifying their spatiotemporal carbon dynamics in western Hunan, thereby providing empirical evidence for guiding sustainable forest management and maximizing climate benefits [6].

Although planted forests have expanded globally and are widely promoted for climate change mitigation, their effectiveness as carbon sinks remains subject to debate. While some studies suggest that plan planted forests tations established on non-forested lands, such as cropland or grassland, can substantially increase aboveground biomass and carbon stocks [7], others report that converting natural into planted forests may lead to lower total ecosystem carbon, particularly due to losses in soil organic carbon and belowground biomass [8, 9]. These inconsistencies may be attributed to a variety of factors, including species composition, stand age, land use change, climatic and geographic conditions [10–14]. For example, planted forests with fast-growing conifers like *Picea abies* and *Pinus ponderosa* often accumulate more belowground biomass than natural forests, whereas planted forests dominated by broadleaved species such as *Populus deltoides* tend to store less [8, 15–17]. Likewise, soil carbon stocks have been found to be lower in planted forests in tropical regions but higher in temperate zones [8, 18, 19]. These context-dependent controversies underscore the critical need for regional, long-term empirical comparisons that move beyond generalized debates. Such comparisons must quantify the actual carbon gains from afforestation relative to natural forests, accounting for specific land-use conversion pathways.​​ Regional, long-term comparisons that consider land-use history and actual carbon gains are needed to clarify their contribution to ecosystem carbon dynamics.

In China, extensive afforestation and forest conservation policies since the 1990s—most notably the Grain-for-Green Program (GGP) and the Natural Forest Protection Program (NFPP)—have profoundly reshaped forest composition and land-use patterns nationwide [20]. The Wuling Mountains region in western Hunan Province, encompassing Zhangjiajie, Huaihua, and Xiangxi Prefecture, represents a typical subtropical mountainous landscape with high forest coverage, complex topography, and substantial ecological heterogeneity [21]. The region covers both well-preserved natural broadleaf forests and widespread planted forests dominated by fast-growing coniferous species such as *Cunninghamia lanceolata* and *Pinus massoniana* [22, 23]. An assessment of ecosystem service value indicates that forest land, being the dominant land cover, contributes over 77% to the total functional magnitude of ecosystem services in the area [24]. In recent decades, the region has undergone rapid land-use transitions, particularly an expansion of timber planted forests, reflecting a policy-driven shift toward more productive forestry practices [25]. These changes have been strongly shaped by national ecological restoration programs and supported by institutional reforms in forest tenure and land management [26]. The coexistence of both extensive natural forests and large-scale planted forests—coupled with diverse forest origins, stand structures, and management histories—makes this region a representative case for assessing whether planted forests can significantly enhance carbon sinks relative to natural forests under dynamic environmental and policy contexts.

In this study, we aim to quantify the contribution of planted forests to total carbon storage increases over a 30-year period in a subtropical region of China. Specifically, we examine: (1) the spatial and temporal changes in natural and planted forest area; (2) the differences in per-hectare carbon storage between the two forest types; and (3) the source land types converted into planted forests and their associated carbon gains. By identifying how land-use transitions have shaped carbon dynamics, this study provides critical insights into the role of afforestation in enhancing regional carbon sinks and informs future land management and climate mitigation strategies.

## Materials and methods

### Study area

**Fig. 1:**
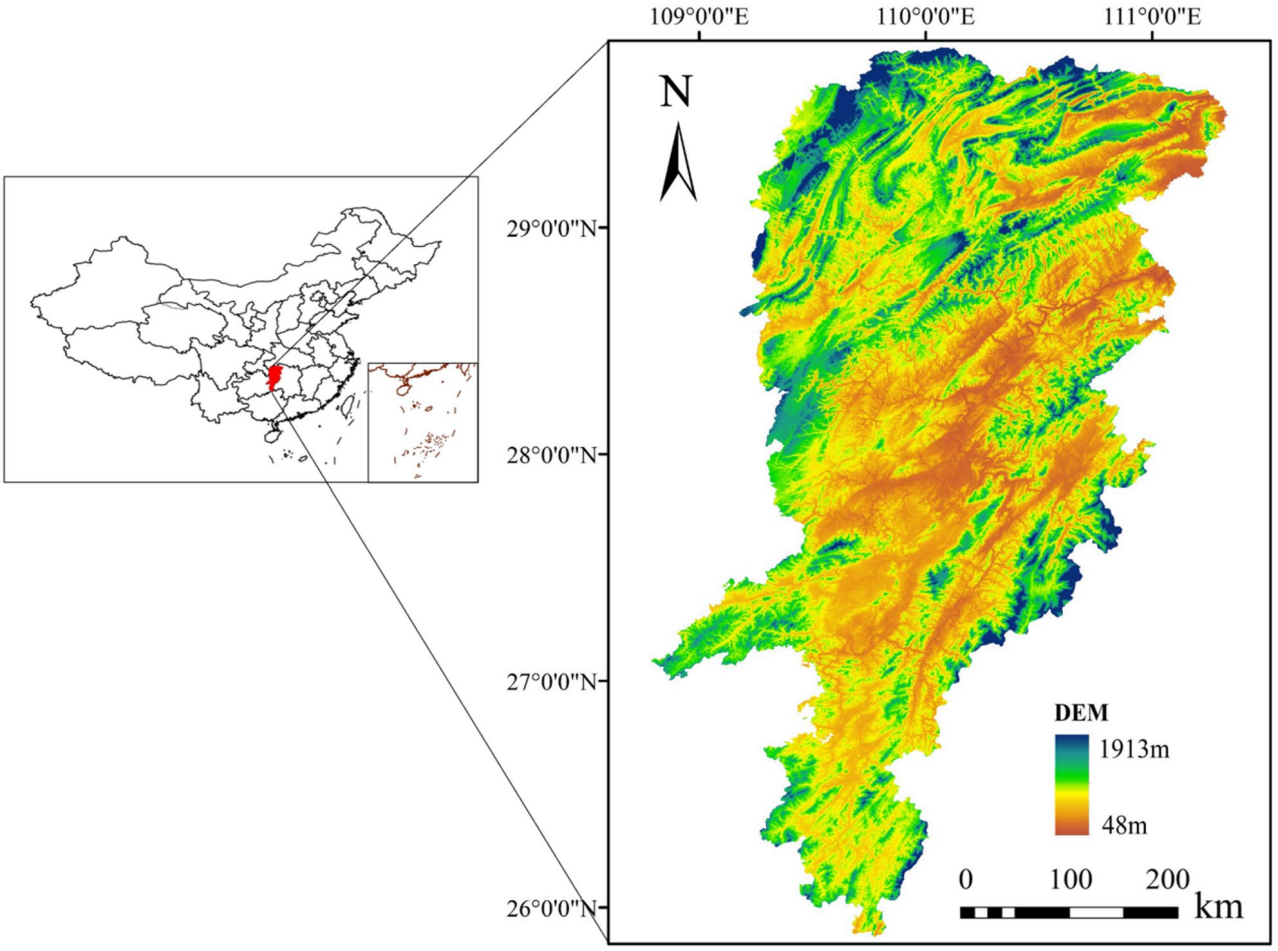
Location, topography, and forest elevation distribution of the study area in western Hunan Province, China. It covers three administrative regions—Zhangjiajie City, Huaihua City, and Xiangxi Tujia and Miao Autonomous Prefecture. The study area features complex terrain with elevations ranging from 48 to 1913 m

The study area is located in the western part of Hunan Province, China, encompassing three administrative regions: Zhangjiajie, Huaihua, and Xiangxi Tujia and Miao Autonomous Prefecture (with Jishou as the capital) [27]. Geographically, it spans from 26° to 29°^。^N and 109° to 111° E, covering a total area of over 38,000 km² (Fig. 1). In addition, the region lies within the Wuling Mountains, a key ecological corridor in subtropical China, and is characterized by rugged topography with elevations ranging from 48 to 1913 m. This complex mountainous landscape supports diverse climatic and ecological conditions and has experienced significant afforestation and land-use change in recent decades. The digital elevation model (DEM) shown in Fig. 1 highlights the strong altitudinal variation, which influences vegetation distribution, microclimates, and ecosystem processes across the region. Notably, planted forests are mainly concentrated between 300 and 800 m, while natural forests are more broadly distributed and dominate higher elevations above 600 m (Fig. 1).

### Datasets

This study uses the annual land use dataset with a 30-meter resolution for China, developed by the Xin Huang et.al at Wuhan University, referred to as the China Land Use Dataset (CLUD) for the past several years [28]. The dataset is based on Landsat series satellite imagery and constructed using the Random Forest classification algorithm, with an overall classification accuracy exceeding 80%. The classification accuracy of forest types (including planted and natural forests) for four key years: 1990, 2000, 2010, and 2020, achieving producer accuracy above 85% for all years. The raw data was obtained from the Zenodo platform (https://zenodo.org/records/5210928), and it was clipped according to the study area boundary for land use change trajectory analysis. The dataset was aggregated using Majority Resampling, a method that prioritizes retaining the most frequent land cover class within the original pixel. This method is particularly suitable for downscaling classification data. Aboveground biomass carbon storage data was sourced from the 1 km resolution China Forest Carbon Pool Spatiotemporal Dynamic Dataset [29]. The carbon storage data used in this study represent aboveground biomass carbon (hereinafter referred to as ‘carbon storage’ for brevity). The dataset integrates MODIS, GLASS Leaf Area Index (LAI), and national forest inventory data, and applies machine learning methods for inversion. The data was obtained from the Figshare platform (10.6084/m9.figshare.21931161.v1) and is used to analyze the spatiotemporal differences in the carbon sink functions of planted forests and natural forests. The distribution data for planted forests and natural forests is based on the 1990–2020 China 30-meter resolution dataset, which provides five-year interval data for the Natural and Planted Forests (PNF) classification [30]. The dataset was constructed through the collaborative inversion of Landsat time-series spectral characteristics and forest management records. The ​total area​ of planted forests derived from this dataset shows a difference of less than 5% when compared to the figures from the national forest resource inventory results, and the provincial statistical yearbook verification shows an R² of 0.82–0.89 (*P* < 0.01).

After obtaining the raw data from GUO-LAB (https://www.3decology.org/2024/04/15/chinas-planted-forest-maps-from-1990-to-2020/), we further integrated forest land ownership data from western Hunan to remove confounding secondary forest areas. This process ultimately led to the extraction of spatiotemporal distribution patterns of planted forests (mainly dominated by Chinese fir and *masson pine*) and natural forests (mainly dominated by evergreen broadleaf forests) within the study area. For the 30-meter resolution PNF dataset, area proportion-weighted resampling was applied: the proportion of planted forest pixels within each 1 km grid was calculated, and grids with ≥ 50% planted forest pixels were marked as planted forest-dominated grids. The 30-meter resolution DEM data used in this study to extract the elevation of the study area was sourced from the Advanced Land Observing Satellite research and application project, available at https://www.eorc.jaxa.jp/ALOS/en/dataset/aw3d30/aw3d30e.htm.

### Methods

This study employs a multi-scale spatiotemporal analysis method, and the research covers the following aspects: changes in area of planted and natural forests, distribution characteristics along elevation gradients, differences in carbon storage, and carbon storage changes driven by land-use changes. All data processing and analysis were conducted using Python 3.12 and ArcGIS 10.5 statistical software.

First, this study quantifies the temporal changes in the area proportion of planted forests and natural forests to reflect the changing trends in the relative importance of planted forests within the regional forest resources over time. To accurately characterize the dynamic features of planted forest expansion, we applied the Theil-Sen non-parametric estimation method to calculate the annual change rate. This method is robust to outliers, making it particularly suitable for handling noise issues that may arise in mountainous data. The specific calculation is as follows:

1 $$\:\begin{array}{c}\beta\:={median}\left(\frac{P\left({t}_{j}\right)-P\left({t}_{ij}\right)}{{t}_{j}-{t}_{i}}\right),\:\:i<j\end{array}$$

P(t_i_) and P(t_j_) represent the area proportion of planted forests (%) in years t_i_ and t_j_, respectively. The time interval is denoted as t_j_ - t_i_ (in years). Significance testing was performed using the Mann-Kendall method (α = 0.05), and pixels with significant changes were displayed in a spatial distribution map. To visually represent the spatial pattern, significant change pixels were marked with black dots on the distribution map. Additionally, the frequency distribution of the change rate was analyzed using Kernel Density Estimation (KDE). Furthermore, the area distribution of planted forests and natural forests was statistically analyzed according to elevation changes. Next, by constructing a land use transition matrix for the period 1990–2020, this study quantified the process of different land cover types transitioning to planted forests. The analysis primarily focused on the sources of planted forest expansion (such as shrubs, grasslands, croplands, etc., converting to planted forests), changes in natural forests (areas converting to planted forests and other land types), and stable regions (forests that remained unchanged). The significance differences were then compared. The calculation is as follows:

2 $$\:\begin{array}{c}C{R}_{i\to\:p}=\frac{{t}_{i,p}}{{\Sigma}_{m-1}^{k}\:{t}_{m,p}}\end{array}$$

CR represents the contribution of each land type to the transition to planted forests, with a focus on calculating the proportion of shrubland, grassland, and cropland converted to planted forests. i→p: represents the specific conversion process from land type i to planted forest (p). t_i, p_ denotes the area converted from land type i to planted forest. The denominator represents the total area of all land types converted to planted forest during the study period. The calculations for stable natural forests and stable planted forests are as follows:

3 $$\:\begin{array}{c}SR=\frac{{t}_{n/p\to\:p/n}}{{A}_{1990}^{n/p}\:}\end{array}$$

SR represents the proportion of stable regions, while n/p correspond to natural forests and planted forests, respectively. A chi-square test was conducted on the carbon storage of each category to determine the significance of the differences.

4 $$\:\begin{array}{c}{x}^{2}=\sum_{i-1}^{k}\frac{{\left({O}_{i}-{E}_{i}\right)}^{2}}{{E}_{i}}\end{array}$$

O_i_ represents the actual observed carbon storage for category i, and E_i_ represents the expected carbon storage for each category (assuming the carbon storage is the same across categories). Additionally, we applied the Getis-Ord Gi* statistic to identify the spatial clustering features of planted forest expansion:

5 $$\:\begin{array}{c}{G}_{i}^{*}=\frac{\sum_{j-1}^{n}{\omega\:}_{ij}-\stackrel{-}{X}\sum_{j-1}^{n}{\omega\:}_{ij}}{\sqrt[S]{\frac{n\sum_{j-1}^{n}{\omega\:}_{ij}^{2}-(\sum_{j-1}^{n}{\omega\:}_{ij}{)}^{2}}{n-1}}}\end{array}$$

x_j_ represents whether pixel j has experienced planted forest expansion (1 = yes, 0 = no), and ω_ij_ is the spatial weight matrix (3 km neighborhood). ‾X represents the arithmetic mean of all observed values x_j_ in the study area. S represents the standard deviation of all observed values x_j_ in the study area. n represents the total number of locations (pixels) in the study area. Significance testing is based on the condition Gi* > 1.96 (*P* < 0.05) for identifying significant hotspots.

Additionally, by integrating land use transition data and carbon storage change information, this study systematically evaluates the impact of different land cover types transitioning to planted forests on carbon storage. Using a 5-year interval, land use change and carbon storage change were analyzed synchronously within each period. To quantify the sensitivity of our results to resampling method selection and ensure robustness, we conducted a systematic sensitivity analysis. We performed 200 iterations of random sampling, each time comparing 50% of randomly selected pixels between the resampled and the original resolution map. This Monte Carlo approach allowed us to assess the accuracy and consistency of different resampling algorithms. Our analysis revealed that Majority Resampling outperformed other methods, yielding the highest mean overall accuracy (0.91) and Kappa coefficient (0.94) against the original classification (Fig. A5 and Table A2). Based on these findings, which demonstrate the superior performance of Majority Resampling in preserving categorical integrity, this method was selected for all subsequent spatial aggregations in our study. The land use dataset was aggregated using ​Majority Resampling, a method that prioritizes retaining the most frequent land cover class within the original pixel. For each pixel, the area corresponding to the transition to planted forests was statistically analyzed, along with its carbon storage change. Pairwise statistical analysis of land use transitions was performed using the *pairwise_tukeyhsd* package from Python 3.12’s library, and Sankey diagrams were used to display the flow of land use and carbon storage changes.

## Results

### Spatial-temporal dynamics of natural and planted forests

**Fig. 2:**
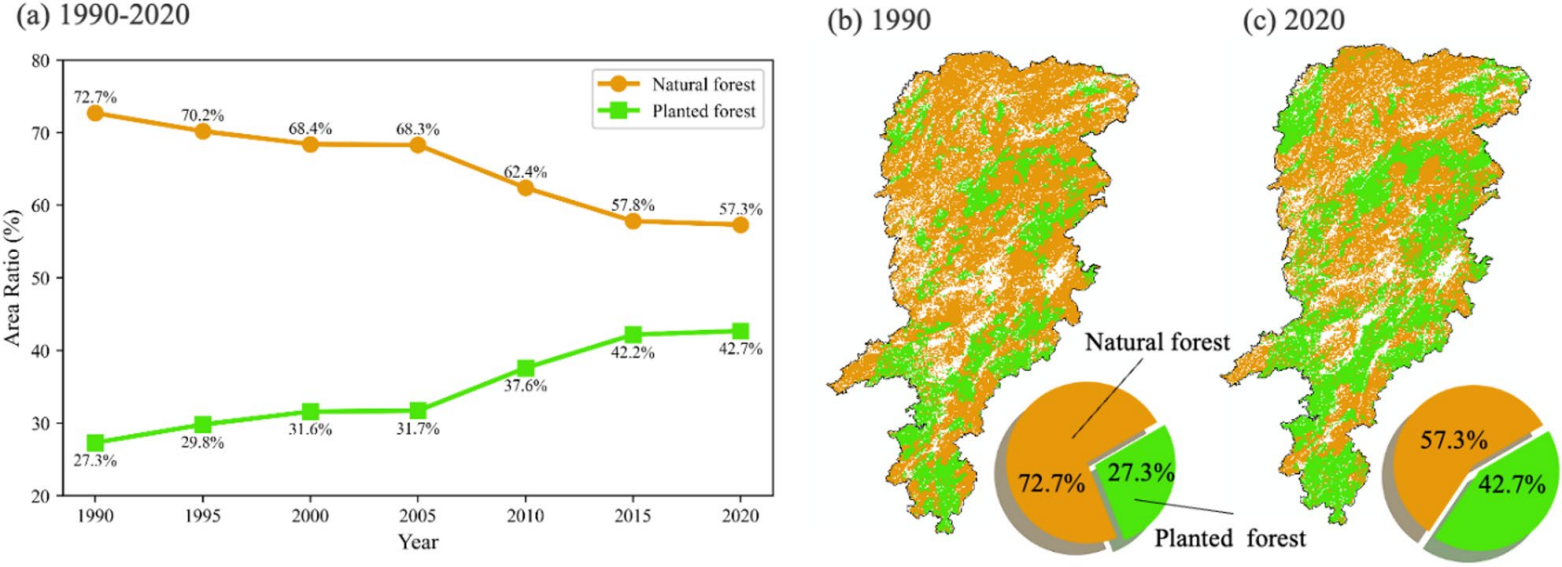
Temporal and spatial changes in the composition of natural and planted forests from 1990 to 2020 in the study area. (**a**) Changes in the area ratio (%) of natural forest and planted forest over the 30-year period. (**b-c**) Spatial distribution of natural forest (orange) and planted forest (green) in 1990 and 2020, respectively. Pie charts indicate the proportion of each forest type in the corresponding year

The composition and spatial distribution of forest types in the study area underwent substantial changes from 1990 to 2020 (Fig. 2). Over this 30-year period, the area proportion of planted forests increased from 27.3% to 42.7%, while that of natural forests declined from 72.7% to 57.3% (Fig. 2a). In 1990, natural forests were widely distributed across the northern, central, and southwestern parts of the region, forming contiguous forest blocks along higher elevations and steeper slopes (Figs. 1 and 2b). In contrast, planted forests were relatively fragmented and mainly concentrated in low-elevation basins and southeastern areas. By 2020, the spatial footprint of planted forests had expanded considerably, occupying large portions of the central and southeastern zones that were previously dominated by natural forest (Fig. 2c). This transition was further confirmed by pixel-based density maps (Fig. A1a, A1b), which showed increasing density of planted forest pixels in these areas and a corresponding reduction in the density and continuity of natural forest pixels. Specifically, the increase in planted forest area appears more pronounced in mid- to low-elevation regions (300–700 m) with relatively gentle terrain, while natural forests consistently occupied larger areas across the entire elevational gradient, particularly between 400 and 900 m in the northern and western parts of the region (Fig. A1c).

**Fig. 3:**
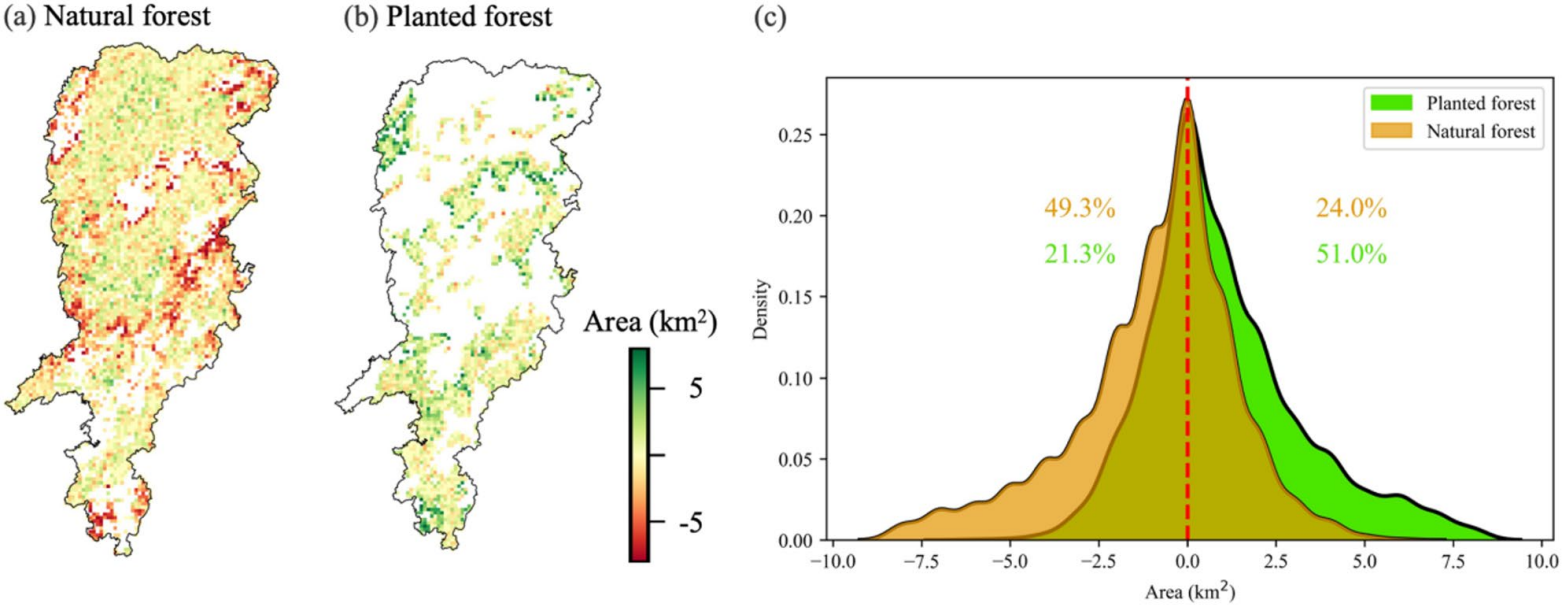
Pixel-based net forest area changes between 1990 and 2020 across the study area. (**a-b**) Spatial distribution of net change in natural forest area and planted forest area, calculated as the difference between 2020 and 1990 at the pixel level. The gradient color from red to green indicates the area of reduction and increase. (**c**) Density distribution curve showing the proportion of pixels with area gain, loss, or no change for both forest types

The pixel-based analysis revealed spatially distinct net changes in forest area from 1990 to 2020, with contrasting patterns between natural and planted forests (Fig. 3). For natural forests, area changes primarily concentrated in the northern and some central parts of the study area (Fig. 3a). Specifically, 24.0% of pixels showed increases, generally forming contiguous clusters in higher elevation zones in the north, while 49.3% of pixels experienced area losses, predominantly scattered across northwest, central and southern subregions (Fig. 3a and c). Temporal trend analysis further revealed that 59.1% of natural forest pixels exhibited statistically significant negative five-year change trends, with notable concentrations of declining trends in the southern, northwestern and some central areas (Fig. A2a; Fig. A2c). In contrast, planted forest area changes were more spatially extensive and uniformly distributed across the region (Fig. 3b). Over the study period, 51.0% of pixels showed net gains in planted forest area, particularly across the central, southern, and northwestern zones (Fig. 3b and c). Meanwhile, 21.3% of pixels indicated area reductions, though these declines were spatially interspersed within expanding regions and not strongly clustered. The five-year trend analysis also showed that 55.9% of planted forest pixels exhibited significant positive trends, with dense distributions of statistically significant pixels (black dots) particularly evident in the central and northwestern areas (Fig. A2b; Fig. A2c). These results collectively indicate a widespread expansion of planted forests and a concurrent decline in natural forests over the study period, with particularly notable trends in the southern subregions, as well as in the northwestern and some central areas.

### Carbon storage changes of natural and planted forests

**Fig. 4:**
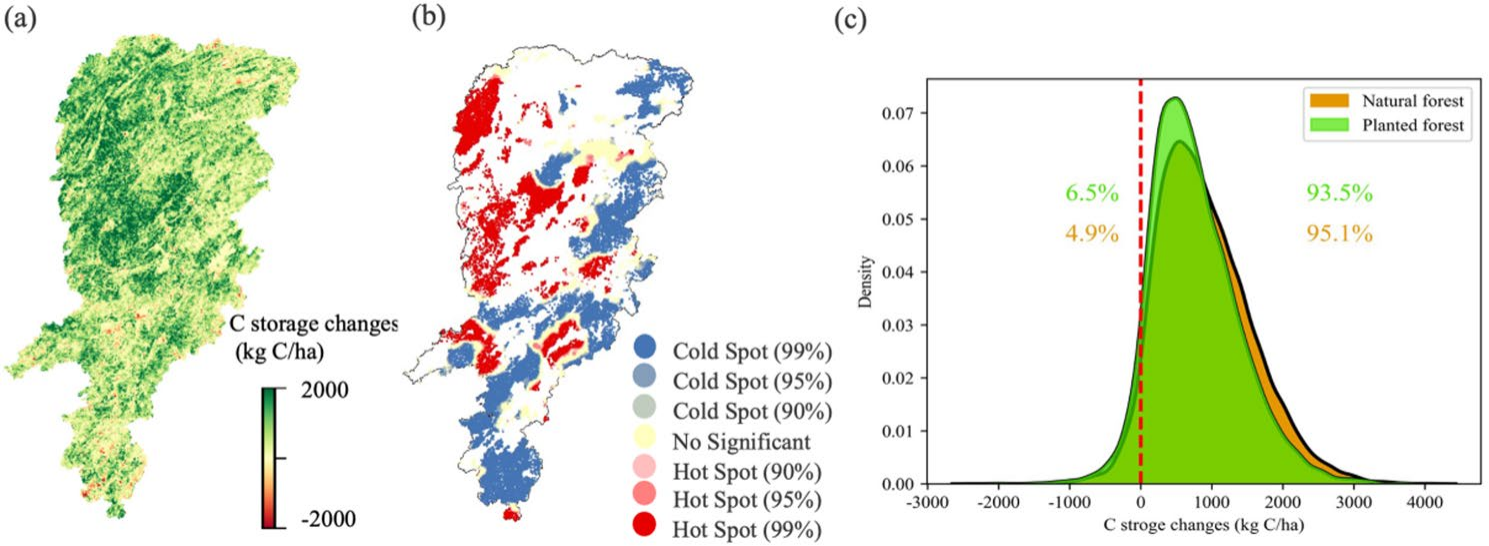
Spatial and statistical characterization of forest carbon storage changes from 1990 to 2020. (**a**) Spatial distribution of carbon storage changes (kg C/ha) in 2020 compared to 1990. (**b**) Hotspot analysis identifying spatial clusters of significantly increased carbon storage areas. (**c**) Density distribution curve showing the proportion of pixels with carbon storage changes in 2020 compared to 1990 for both forest types

Figure 4 illustrates the spatial patterns of carbon storage changes between 1990 and 2020 for both natural and planted forests across the study region. At the pixel level, most areas showed substantial increases in carbon storage (kg C/ha) in 2020 compared to 1990, with widespread gains observed in both natural (95.1% of pixels) and planted forests (93.5% of pixels), particularly concentrated in the central, western, and northwestern subregions (Fig. 4a and c). Localized declines were mainly found in parts of the southern subregions (Fig. 4a). These patterns were further supported by the density distributions, which showed that 95.6% of planted forest pixels and 96.5% of natural forest pixels experienced consistent increases in carbon storage across consecutive five-year intervals (Fig. A3b). Most statistically significant gains (black dots) were clustered in the central and northwestern regions (Fig. A3a). Hotspot analysis revealed that high-intensity carbon accumulation zones (red hotspots) were primarily located in the central, western, and northwestern areas, whereas cold spots (blue), indicating relatively slower carbon increases, were mainly distributed in the eastern and southern subregions (Fig. 4b).

**Fig. 5:**
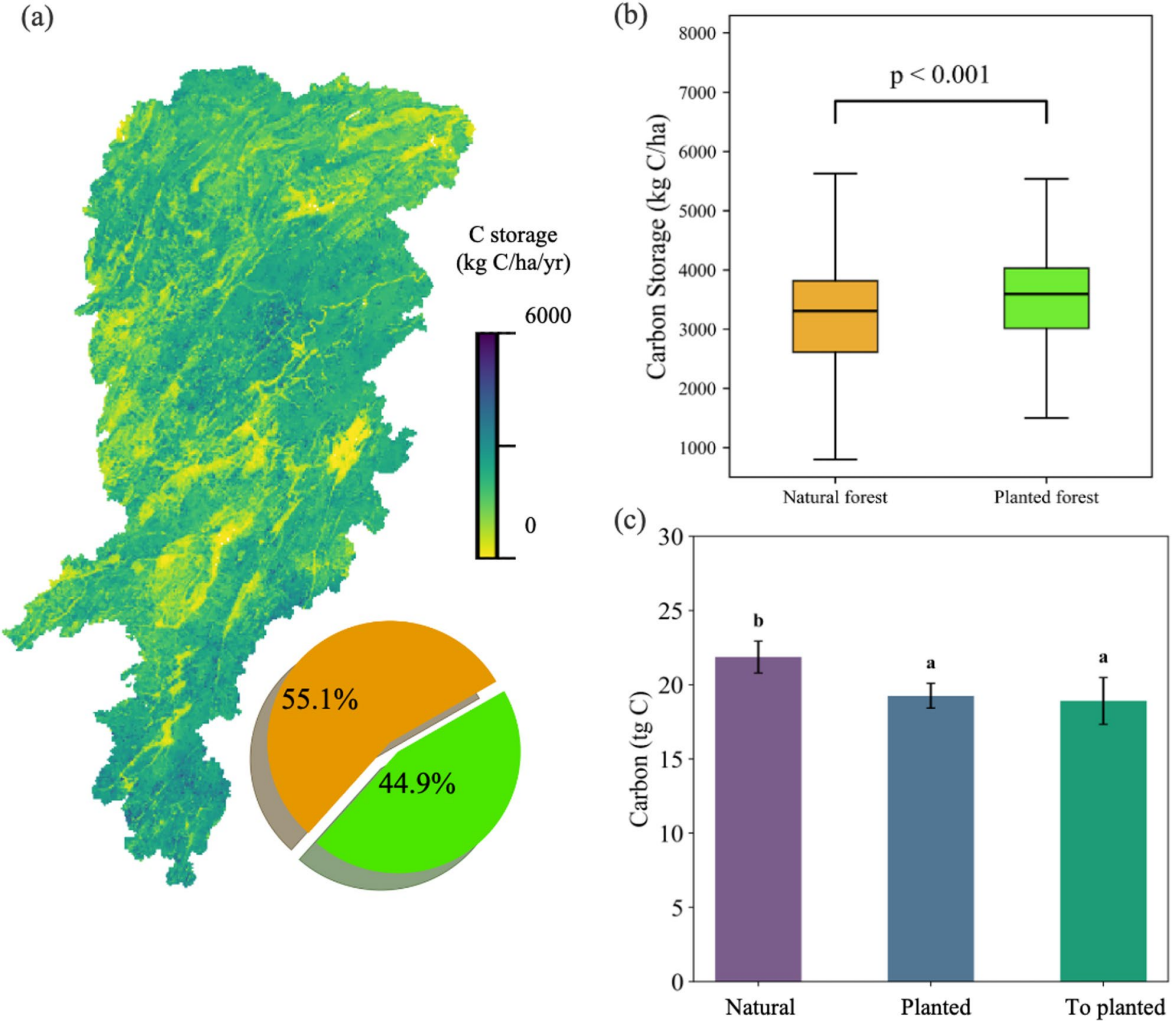
Spatial distribution and comparison of mean annual forest carbon storage between natural and planted forests. **(a**) The spatial distribution of mean annual carbon storage (kg C/ha/year) across the study region, with the accompanying pie chart indicating the proportional contributions of natural and planted forests. (**b**) carbon storage per unit area (kg C/ha) between natural and planted forests. (**c**) Total carbon storage (Tg C) in stable natural forests (“Natural”), stable planted forests (“Planted”), and forests that transitioned from other land use types to planted between 1990 and 2020 (“To planted”). The ‘a’ and ‘b’ in the figure indicate significant differences between groups

The spatial distribution of forest carbon storage changes over the 30-year period exhibited clear differences between natural and planted forests across the study region (Fig. 5). On average, natural forests contributed 55.1% of the total annual carbon storage (kg C/ha/year), while planted forests accounted for the remaining 44.9%, as shown in the pie chart in Fig. 5a. The accompanying spatial map highlights those areas with high carbon storage were primarily concentrated in the central, northwestern, and southwestern parts of the region, whereas lower values were observed in the eastern and southern zones. At the pixel level, planted forests demonstrated significantly higher carbon storage per hectare than natural forests (*p* < 0.001), and their distribution exhibited a more concentrated interquartile range, indicating more uniform carbon accumulation patterns (Fig. 5b). In addition, the annual carbon storage per unit area for both forest types showed a continuous upward trend over the study period, as illustrated in Fig. A4.

Carbon storage patterns also varied with forest type history over the 30-year period (Fig. 5c). Natural forests that remained unchanged from 1990 to 2020 stored the highest total carbon, approximately 23.0 Tg C. Stable planted forests, those classified as planted in both 1990 and 2020, stored about 19.5 Tg C. In contrast, forests that transitioned from other land use types to planted types over this period (“to planted”) contributed about 19.0 Tg C, indicating a near doubling of carbon stocks within these newly established or converted planted forests.

### Land-use conversion and its carbon contributions

**Fig. 6:**
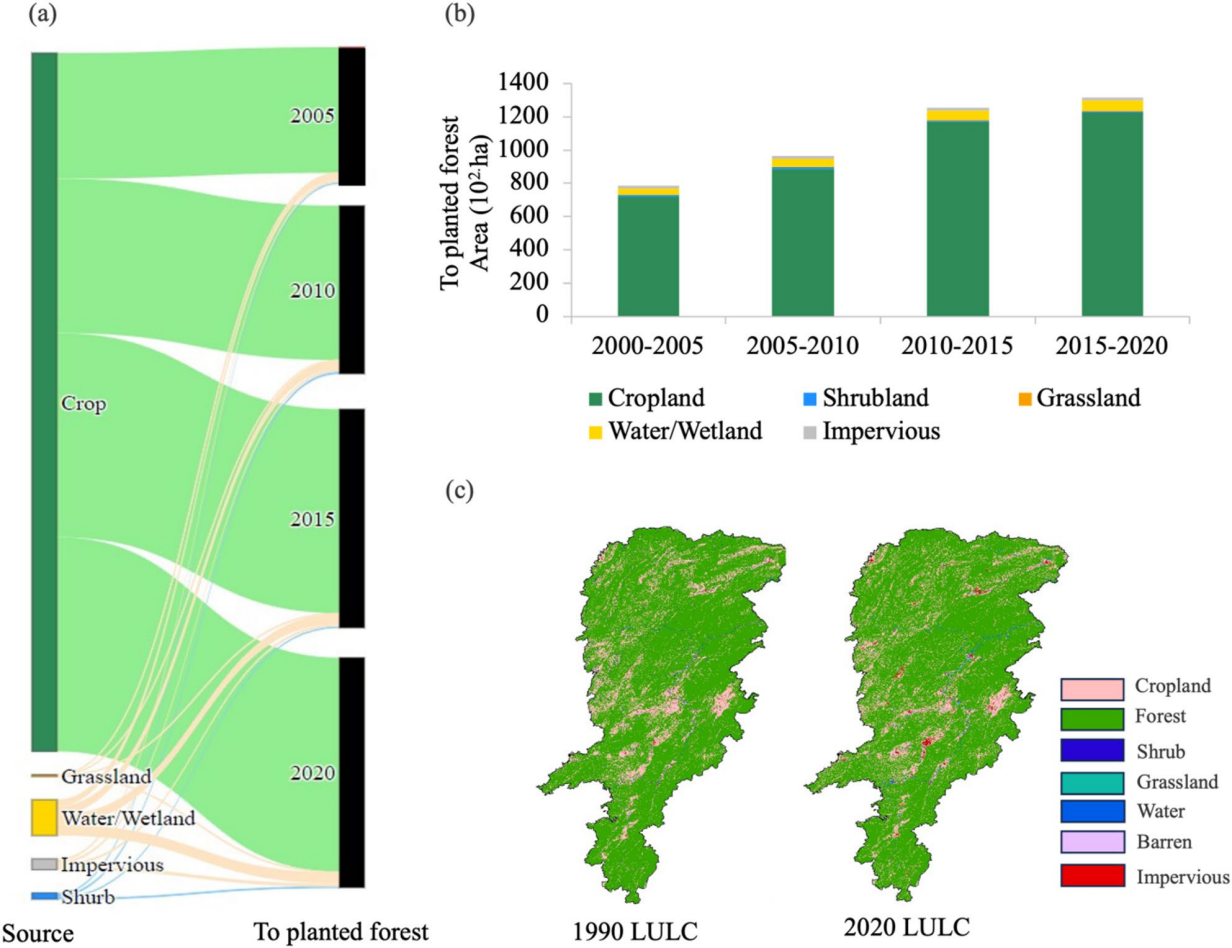
Land-use transitions and spatial patterns of planted forest expansion from 1990 to 2020. (**a**) Sankey diagram showing the sources of land converted to planted forests at four time points (2005, 2010, 2015, and 2020). The thickness of the flow lines indicates the area contribution of each land-use type. (**b**) Statistical area of different land use types converted to planted forests at four time points (**c**) Spatial distribution maps of land-use classes in 1990 and 2020

The Sankey diagram (Fig. 6a) reveals cropland as the primary contributor to newly established planted forests across all four periods (2005, 2010, 2015, and 2020). This dominance is quantitatively supported by the stacked bar chart (Fig. 6b), where cropland (green bars) consistently accounts for the largest share of conversions. Notably, the total area converted from cropland increased markedly over time: rising from 721 × 10² ha in 2000–2005 to 1,229 × 10² ha in 2015–2020 (Fig. 6b; Table. A1). This transition pathway reflects widespread agricultural land abandonment or planned afforestation policies implemented over the past two decades. In addition to cropland, other land types such as grassland, shrubland, wetlands, and impervious surfaces also contributed to planted forest expansion, albeit to a much lesser extent. Among these, water or wetlands provided the second-largest area of conversion, with their area converted to planted forest increased from 38 × 10^2^ha during 2000 to 2005 periods to 63 × 10^2^ha during 2015 to 2020 periods (Fig. 6b; Table. A1).

Between these two periods, notable increases in planted forest area are clearly visible, particularly in areas where cropland (light pink) was previously dominant (Fig. 6c). The most significant expansions occurred in the central, western, northwest northeast, and southeastern parts of the region, where extensive patches of pink land cover transitioned to green, indicating forest establishment. Additional transitions are scattered in the eastern and southern areas as well.

**Fig. 7:**
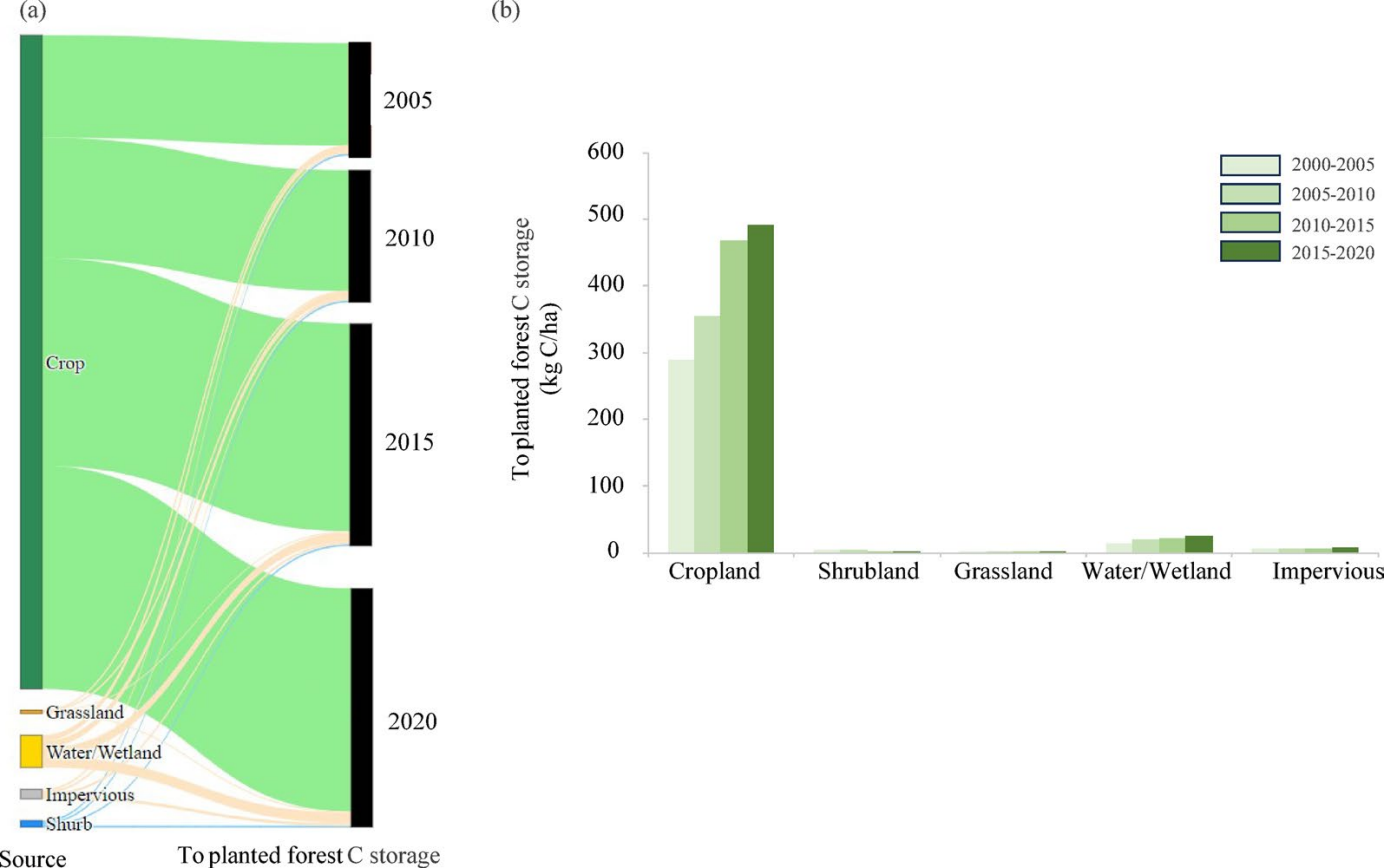
Carbon sequestration contributions from different land cover sources converted to planted forests over time (2000–2020). (**a**) Sankey diagram showing the trajectories of carbon storage resulting from various land-use conversions to planted forests at four time points. (**b**) Carbon storage per hectare in planted forests established on different land covers

The Sankey diagram in Fig. 7a shows the trajectories of carbon storage resulting from various land-use conversions to planted forests across four time points (2005, 2010, 2015, and 2020). Throughout the entire period, cropland consistently emerged as the largest contributor to carbon storage through conversion to planted forests, as evidenced by the visually dominant flow lines at each of the four-time nodes (Fig. 7a). We further reveals that planted forests established on cropland consistently exhibited the highest carbon storage per hectare across all four years (Fig. 7b). The carbon storage increased over time, from just 288.4 kg C/ha during the initial five years (2000–2005) to nearly 491.6 kg C/ha in the last five years (2015–2020) (Table. A1). In contrast, planted forests established on water/wetlands, though significantly lower than cropland, displayed the second-highest carbon storage per hectare, ranging from 15.2 kg C/ha (2000–2005) to 25.2 kg C/ha (2015–2020). The carbon sequestration from conversions of impervious surfaces, grasslands, and shrublands remained minimal, with comparatively narrower flow lines across all time intervals, consistently staying below 8 kg C/ha throughout the period (Fig. 7b; Table. A1).

## Discussion

### Spatiotemporal expansion of planted forests and decline of natural forests

Our study points to a gradual yet accelerating temporal trend in plantation establishment, driven by the need for sustainable resource management and environmental solutions (Meng et al., 2023; Wang et al., 2025). This trend aligns with global shifts in forest management, where reforestation and afforestation efforts have been prioritized as part of climate change mitigation strategies [31, 32]. However, the observed net expansion of planted forests—primarily onto former croplands as identified in our land-use conversion analysis—highlights a nuanced reality. It underscores the success of ecological restoration policies in enhancing regional carbon sinks without directly causing large-scale deforestation. Yet, the concurrent decline in natural forest area, particularly in certain subregions, ​demands a more cautious evaluation. This pattern implies that indirect pressures, such as the displacement of agricultural activities or increased fragmentation, may pose threats to natural ecosystems. Therefore, while the expansion of planted forests aligns with sustainable resource management goals, our results emphasize that its net environmental benefit depends critically on careful land-use planning that safeguards existing natural forests [33, 34].

The spatial distribution of these changes further underscores distinct ecological niches. We observed that planted forests have expanded predominantly in mid- to low-elevation regions (300 to 700 m), where the terrain is more conducive to plantation establishment, while natural forests have largely persisted in higher-elevation zones (400 to 900 m). This spatial variation reflects the differential ecological requirements of each forest type: natural forests often occupy more topographically complex and less accessible terrain, which limits large-scale plantation development [35–37]. Geographically, the central, southern, and northwestern regions of western Hunan marked land-use occurred, with natural forests increasingly replaced by planted forests. These changes initially appeared as fragmented patches, but by 2020 had expanded into more continuous areas, particularly in the central and southeastern zones. Such patterns highlight the importance of integrated, strategic land-use planning to reconcile ecological objectives with production needs in western Hunan province [38, 39]. As emphasized in a recent EU-wide assessment, achieving biodiversity and climate mitigation goals without undermining food and timber security requires careful coordination of restoration, conservation, and land-use demands across spatial and policy scales [40]. This insight ​finds a potent illustration in the Chinese context, particularly within the framework of its “Ecological Civilization” strategy.​​ Here, the tension manifests as a competition for land between ​strictly protected zones (e.g., ecological conservation redlines) and areas designated for economic development, such as the ecotourism hubs within our study area.​​ Our findings underscore the critical importance of China’s ongoing efforts in ​​"multi-planet integration"​​ —coordinating ecological, agricultural, and urban development boundaries—to implement the nuanced land-use planning this insight advocates [39, 41].

### Planted forests have higher carbon accumulation efficiency

Our study demonstrates a critical distinction in carbon dynamics between forest types: while natural forests constitute the larger carbon reservoir (contributing 55.1% of total annual storage), planted forests exhibit significantly higher carbon sequestration efficiency per unit area. This finding underscores their specialized role in rapid carbon capture. The efficiency of planted forests can be largely attributed to the selection of fast-growing species and intensive management practices, which optimize growth conditions and biomass accumulation [22, 42]. In contrast, the growth dynamics in natural forests are more complex and variable, often resulting in lower average annual sequestration rates per hectare [43, 44].

Our findings reveal a complementary role of natural and planted forests in the regional carbon budget: natural forests serve as large, stable carbon stocks, while planted forests act as efficient carbon sequestration agents. As expected, natural forests that persisted from 1990 to 2020 formed the largest carbon pool (23.0 Tg C), reflecting centuries of accumulated biomass. Stable planted forests stored 19.5 Tg C [43]. The most compelling evidence for the efficiency of planted forests, however, comes from the areas converted to planted forests during the study period, which accumulated nearly equivalent carbon stocks (19.0 Tg C) to the stable planted forests [22]. This near-doubling of carbon in these converted areas highlights the remarkable potential of afforestation to rapidly establish significant carbon sinks on formerly non-forested or degraded lands [45].

The rapid carbon accumulation in newly planted forests highlights the role of planted forests in restoring degraded lands and contributing to carbon sequestration. The total carbon storage in natural forests that remained unchanged from 1990 to 2020 was higher than in stable planted forests, with natural forests storing 23.0 Tg C compared to 19.5 Tg C in stable planted forests. This reflects the long-term carbon accumulation in natural forests [46]. However, a striking observation was that forests transitioning from other land-use types to planted forests during the study period stored nearly as much carbon as stable natural forests. This near doubling of carbon stocks in converted areas emphasizes the significant potential of afforestation and reforestation efforts to enhance carbon storage, particularly in degraded landscapes [47]. Therefore, these findings underscore the importance of land-use policies that promote reforestation and afforestation to complement the carbon storage contributions of natural forests [48]. However, long-term monitoring is essential to ensure that planta planted forests tions continue to function effectively as carbon sinks and do not replace more biodiverse natural ecosystems that provide broader ecological benefits [49]. Our results demonstrate the complementary roles of natural and planted forests: natural forests serve as larger carbon stocks that must be conserved to prevent emissions, while planted forests act as more efficient carbon sinks that can rapidly sequester atmospheric CO₂ on suitable lands. Confusing these two concepts could lead to misguided policies, such as converting natural forests for planted forests, which would result in a net carbon loss.

### Cropland as the primary source of planted forest expansion and carbon sequestration

Our spatiotemporal analysis revealed a significant expansion of planted forests, characterized by net increases across 51.0% of the study area’s pixels.​​ This large-scale transformation ​can be primarily linked to​ national ecological restoration policies, with the GCP playing a particularly pivotal role in facilitating the conversion of cropland to planted forests. This process, possibly linked to the effective implementation of the “Grain to Green” policy, highlights cropland as the dominant source driving both the establishment of new planted forests and the enhancement of carbon storage [20]. The substantial increase in cropland conversions—from 721 × 10² ha in 2000–2005 to 1,229 × 10² ha in 2015–2020—demonstrates the accelerating momentum toward large-scale afforestation. This trend is especially evident in the central, western, and southeastern regions (Ji shou and Huai Hua city), where land-use change has had a big impact on ecosystem services in recent decades (Chen et al., 2023). By repurposing agricultural landscapes for ecological restoration, cropland has become an efficient resource for expanding planted forests and enhancing carbon storage, consistent with previous findings that identified cropland-to-planted forest conversion as a dominant land change under China’s GCP [50]. These conversions emphasize the significant role of cropland in reforestation and afforestation, providing an important avenue for increasing carbon sequestration capacity and helping mitigate the impact of land degradation. By explicitly tracing the carbon consequences of the dominant land-use change (cropland conversion) in our study region, we provide empirical evidence for a ‘managed’ carbon sink that is often poorly constrained in large-scale models.This underscores the importance of integrating spatially explicit land-use history into regional carbon accounting frameworks [51, 52].

Our findings on carbon gains from wetland conversion highlight a critical ecological trade-off. While it may contribute to carbon quotas, this practice leads to the irreversible loss of vital wetland functions. Therefore, policy must strictly prohibit afforestation on natural wetlands and prioritize their conservation and restoration over carbon sequestration objectives. This transition reflects a shift where many wetland areas have been converted into either planted or secondary forests, which, although enhancing carbon sequestration, also suggests the loss of valuable wetland ecosystems [53]. Given the crucial ecological functions that wetlands provide, including biodiversity conservation and water regulation, there is a need to balance the goals of increasing carbon storage with the protection of wetland ecosystems [54]. Research and policy interventions should aim to strike a balance between afforesting wetlands to sequester carbon and preserving their vital ecosystem services [55, 56].

Consequently, future land-use policies must transition from generalized afforestation targets to a ​spatially explicit management framework that applies differentiated land management strategies. This framework should ​categorically prioritize afforestation on marginal or degraded cropland as a high-efficacy carbon sequestration strategy, while ​elevating the conservation and restoration of natural wetlands and existing natural forests to non-negotiable priorities​ to safeguard their irreplaceable ecological functions. The core objective of strategic planning, therefore, shifts from merely maximizing carbon benefits to ​orchestrating land-use transitions that systematically align carbon sequestration goals with the broader imperative of maintaining and enhancing overall ecological integrity.

### Limitations and future perspectives

This study has several limitations that should be considered when interpreting the results. First, the findings of this study are based on remote sensing interpretation and classification data. Although the datasets used has been validated and has high accuracy, factors such as shadows and phenological changes under complex mountainous terrain still introduce classification uncertainties. Additionally, resampling the data to 1 km for multi-source data fusion, although using a majority resampling method to maximize the preservation of category information and minimize noise, will still smooth out small patches. Future studies could employ higher-resolution imagery (e.g., Sentinel-2) combined with object-based classification and advanced fusion techniques (e.g., deep learning-based super-resolution)​​ to better capture fine-scale spatial dynamics and improve classification accuracy in heterogeneous mountainous regions.

Second, the lack of spatially explicit forest stand age data prevents a direct disentanglement of age effects from the inherent growth characteristics of different forest types. While our landscape-scale comparison provides robust evidence of the realized carbon outcomes under real-world conditions (a mix of ages), future studies incorporating age data from forest inventories or remote sensing (e.g., using time since disturbance derived from Landsat time series) could provide a more mechanistic understanding of the carbon accumulation trajectories. ​Future work should integrate forest inventory data or leverage remote sensing techniques to derive forest age (e.g., using Landsat time-series to map time since establishment or disturbance)​. This would allow for a direct comparison of age-specific growth rates between planted and natural forests and more robust projections of long-term carbon sink potential. In addition, our carbon storage data primarily reflect aboveground biomass carbon; including soil organic carbon dynamics would offer a more comprehensive assessment of the ecosystem carbon balance. ​We recommend that subsequent research explicitly quantify changes in SOC stocks following afforestation, particularly on converted croplands, and assess the trade-offs or synergies between above- and belowground carbon pools.​​ This is critical for evaluating the net climate mitigation benefit of land-use changes.

## Conclusions

This study quantified the contribution of afforestation to the regional carbon sink in western Hunan, China, over three decades. Through quantitative statistical and spatial analysis methods, this study aims to reveal the impact mechanisms of planted forest expansion on regional carbon cycling. Our results demonstrate that planted forests, particularly on converted croplands, can rapidly enhance carbon storage, thereby playing a significant role in regional climate mitigation strategies. However, the concurrent decline in natural forest area presents a complex carbon management trade-off that requires careful spatial planning. The significant contribution of cropland conversions to both planted forest expansion and carbon accumulation underscores the potential of afforestation policies, such as the “Grain to Green” initiative, to enhance carbon storage. Additionally, although wetlands have played a secondary role in planted forest expansion, their conversion raises concerns about ecosystem service loss, highlighting the need for a balanced approach to land-use management. Moving forward, it is crucial for policies to integrate afforestation efforts with wetland conservation, ensuring that land-use changes contribute to both carbon sequestration and biodiversity preservation. These findings underscore the importance of adopting sustainable land management strategies to optimize carbon storage and mitigate the impacts of climate change.

## Supplementary Information

Supplementary Material 1

## Data Availability

No datasets were generated or analysed during the current study.
