# Supplementary material for: The contribution of planted forests to regional carbon storage: evidence from western Hunan, China (1990–2020)
Source: Carbon Balance Manag. 2026 Apr 20;21:92. doi: 10.1186/s13021-026-00443-3 (PMC13312525; doi:10.1186/s13021-026-00443-3)
Supplement: Supplementary file 1 — Supplementary Material 1 [file 13021_2026_443_MOESM1_ESM.docx]

**
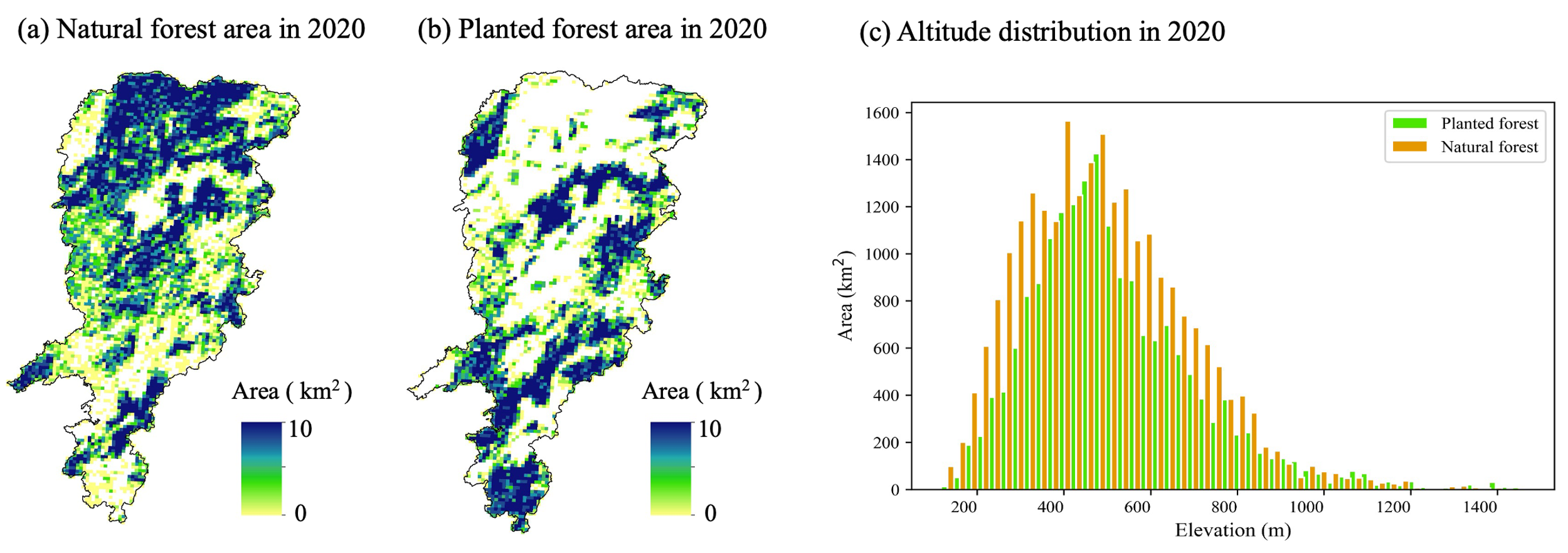
**

**Fig. A1**. Supplementary analysis of spatial changes in forest distribution patterns. (a to b) Pixel-based density maps showing the spatial distribution intensity of natural forest and planted forest in 2020, respectively (darker colors represent higher forest area within each pixel). (c) Elevation-based distribution of natural and planted forests in 2020.


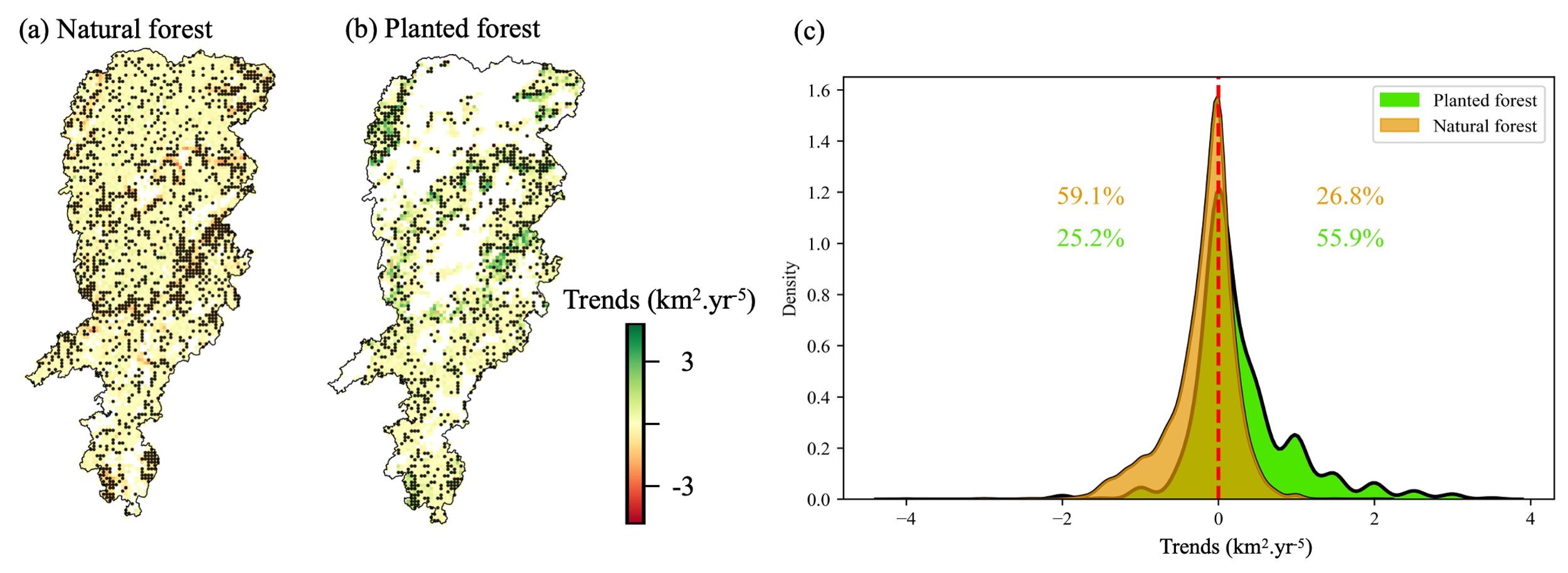


**Fig. A2.** Trends in five-year forest area change rates from 1990 to 2020. (a-b) Spatial distribution of the slope values derived from pixel-level linear regressions of forest area change over successive five-year intervals. Black dots indicate pixels where the temporal trend is statistically significant (p < 0.05). (c) Density distribution curve of slope values for natural and planted forests.

Fig. A3. Trends in five-year carbon storage change rates across the study region from 1990 to 2020. (a) Spatial distribution of the slope values derived from pixel-level linear regressions of carbon storage change over successive five-year intervals. Black dots indicate pixels where the temporal trend is statistically significant (p < 0.05). (b) Density distribution curve of carbon storage change rates for natural and planted forests.


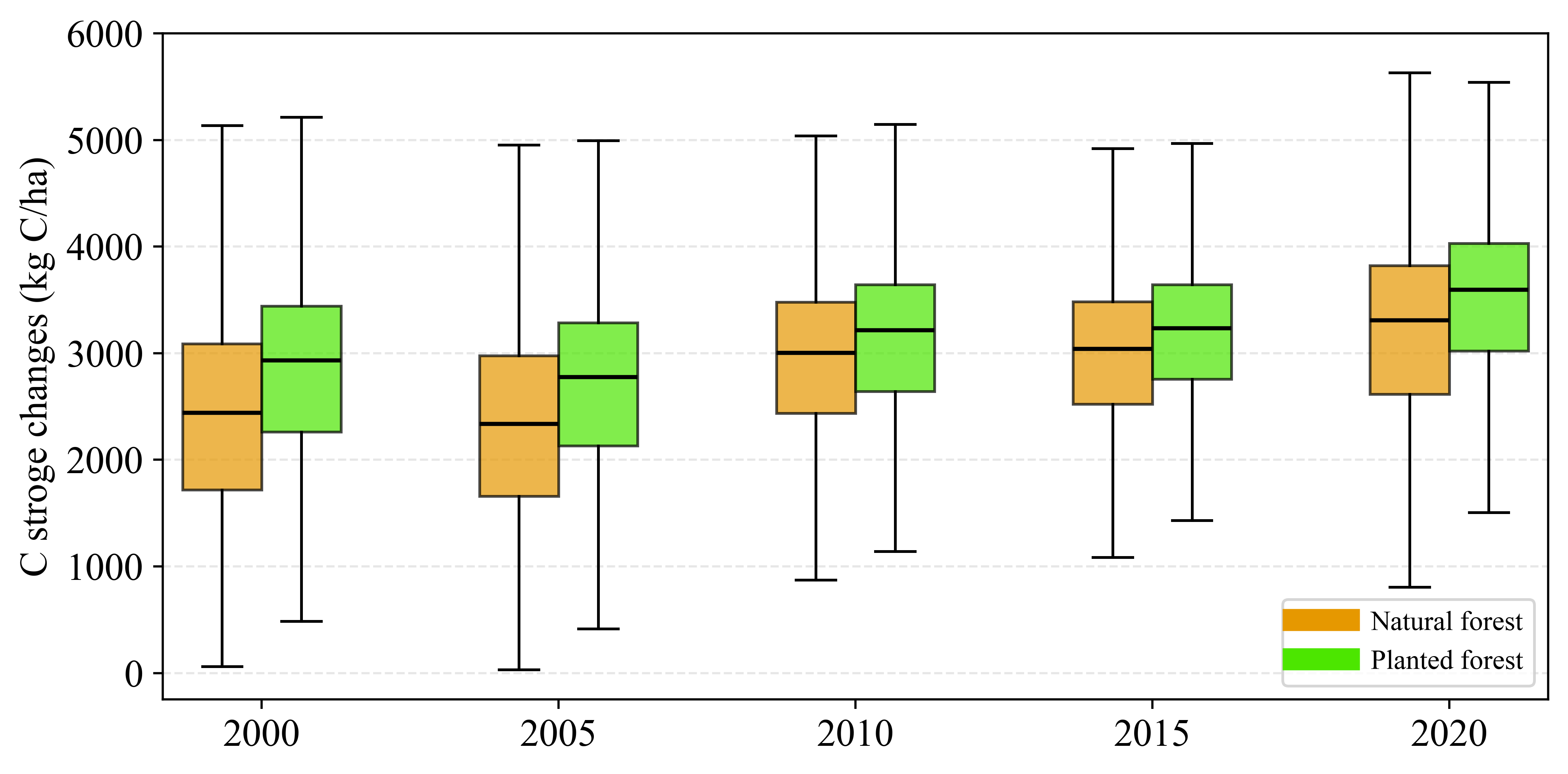


Fig. A4. Carbon storage changes per unit area from 1990 to 2020.

Table. A1. Changes in five-year area and carbon storage from other land use types to planted forest.

| Name | Year | 2000-2005 | 2005-2010 | 2010-2015 | 2015-2020 |
| --- | --- | --- | --- | --- | --- |
| Area to planted forest (10^2^ ha) | Cropland | 721 | 885 | 1169 | 1229 |
|  | Shrubland | 10 | 13 | 9 | 4 |
|  | Grassland | 1 | 2 | 4 | 4 |
|  | Water/Wetland | 38 | 49 | 56 | 63 |
|  | Impervious | 14 | 15 | 15 | 19 |
| To planted forest C storage (kg C/ha) | Cropland | 288.4 | 354 | 467.6 | 491.6 |
|  | Shrubland | 4 | 5.2 | 3.6 | 1.6 |
|  | Grassland | 0.4 | 0.8 | 1.6 | 1.6 |
|  | Water/Wetland | 15.2 | 19.6 | 22.4 | 25.2 |
|  | Impervious | 5.6 | 6 | 6 | 7.6 |
